# Optimization of fuzzy c-means (FCM) clustering in cytology image segmentation using the gray wolf algorithm

**DOI:** 10.1186/s12860-022-00408-7

**Published:** 2022-02-15

**Authors:** Maryam Mohammdian-khoshnoud, Ali Reza Soltanian, Arash Dehghan, Maryam Farhadian

**Affiliations:** 1 Department of Biostatistics, School of Public Health and Research Center for Health Sciences, Hamadan University of Medical Sciences, Hamadan, Iran; 2 Modeling of Noncommunicable Diseases Research Center, Hamadan University of Medical Sciences, Hamadan, Iran; 3 Department of Pathology, School of Medicine, Hamadan University of Medical Sciences, Hamadan, Iran

**Keywords:** Computer-aided diagnosis, Breast cancer, Image segmentation, Machine learning, Gray wolf optimization, Fuzzy c-means, Optimization

## Abstract

**Background:**

Image segmentation is considered an important step in image processing. Fuzzy c-means clustering is one of the common methods of image segmentation. However, this method suffers from drawbacks, such as sensitivity to initial values, entrapment in local optima, and the inability to distinguish objects with similar color intensity. This paper proposes the hybrid Fuzzy c-means clustering and Gray wolf optimization for image segmentation to overcome the shortcomings of Fuzzy c-means clustering. The Gray wolf optimization has a high exploration capability in finding the best solution to the problem, which prevents the entrapment of the algorithm in local optima. In this study, breast cytology images were used to validate the methods, and the results of the proposed method were compared to those of c-means clustering.

**Results:**

FCMGWO has performed better than FCM in separating the nucleus from the other dark objects in the cell. The clustering was validated using Vpc, Vpe, Davies-Bouldin, and Calinski Harabasz criteria. The FCM and FCMGWO methods have a significant difference with respect to the Vpc and Vpe indexes. However, there is no significant difference between the performances of the two clustering methods with respect to the Calinski-Harabasz and Davies-Bouldin indices. The results indicate the better efficacy of the proposed method.

**Conclusions:**

The hybrid FCMGWO algorithm distinguishes the cells better in images with less detail than in images with high detail. However, FCM exhibits unacceptable performance in both low- and high-detail images.

## Background

Image segmentation is the division of an image into discrete regions such that the pixels inside each region have the highest similarity and those across different regions have the highest contrast [1]. Threshold-based, edge-based, region-based, matching-based, clustering-based segmentation, segmentation based on fuzzy inference and generalized principal component analysis are image segmentation techniques [2]. Each of these methods has advantages and disadvantages. Consequently, none of them can be considered a comprehensive image segmentation algorithm [2]. Image segmentation can be considered a classification problem. Hence, machine-learning-based classification algorithms can be of great help in this area [3]. Unsupervised and supervised Learnings are two completely different areas in the spectrum of machine learning and pattern recognition methods. In supervised segmentation, a set of pixel-level images and labels are used, and the goal is to train a system that classifies known class labels for image pixels. The disadvantage of supervised learning techniques is that models are limited to learning from labeled datasets which are often expensive, time-consuming, and sometimes difficult to produce. This issue is more acute in the medical image processing field because the producing high quality datasets requires the effort of experienced and skilled human observers. On the other hand, in ground truth, the accuracy of the assessment depends on two important factors. First, one needs to design or have a proper ground truth, and second, one needs to choose appropriate similarity criteria for the problem being considered. A popular technique is to compare automated techniques with a group of human experts. In this context, one assumed that human evaluators have prior knowledge of ground truth, which is reflected in their manual tracing. Unfortunately, human evaluators may make mistakes and considerations of accuracy and variability must be taken into account. After creating ground truth, the main task of evaluation is to measure the similarity between automatic segmentation and reference. It is yet unclear whether a set of general measurements can be used for all segmentation problems.

Unlabeled datasets require less human effort to create and are easier to obtain. Also, in unsupervised classification, an image is divided into as many meaningful areas as possible without any prior knowledge. In the unsupervised method, there are no training images or ground truth labels of pixels beforehand. Therefore, the number of unique cluster labels must be consistent with the image content.

Fuzzy c-means clustering methods have great potential to extracting detailed features from image pixels. Fuzzy c-means (FCM) clustering is one of the important unsupervised learning algorithms. It requires knowledge of the initial details of some of the parameters, such as the number of clusters and the position of the centroid of the clusters, and its performance depends on the input parameters. Some researchers proposed various methods for estimating the number of clusters or cluster centroids [4, 5]. Moreover, FCM is sensitive to noise and entrapment in local optima. Various metaheuristic methods have been used to optimize the objective function of the fuzzy algorithm in order to avoid entrapment in local optima. Also, FCM fails in distinguishing objects with similar color intensity in images on its own. To overcome the mentioned issues, the Gray wolf optimization (GWO) was used for optimization in this research [6]. The combined use of FCM and GWO to find the optimal cluster centers improves the cluster performance. The main criterion for selecting the best algorithm for medical images is the accuracy of the algorithms. Reducing complexity is the next goal in medical image processing. Therefore, the present study aims to combine FCM with the GWO. Using this combination prevents entrapment in local optima and better optimizes the cluster centers obtained from FCM. In addition, the clustering will be more capable of distinguishing the nucleus from the cytoplasm and other dark-colored cell features in breast cancer cytology images.

## Results

**Fig. 1:**
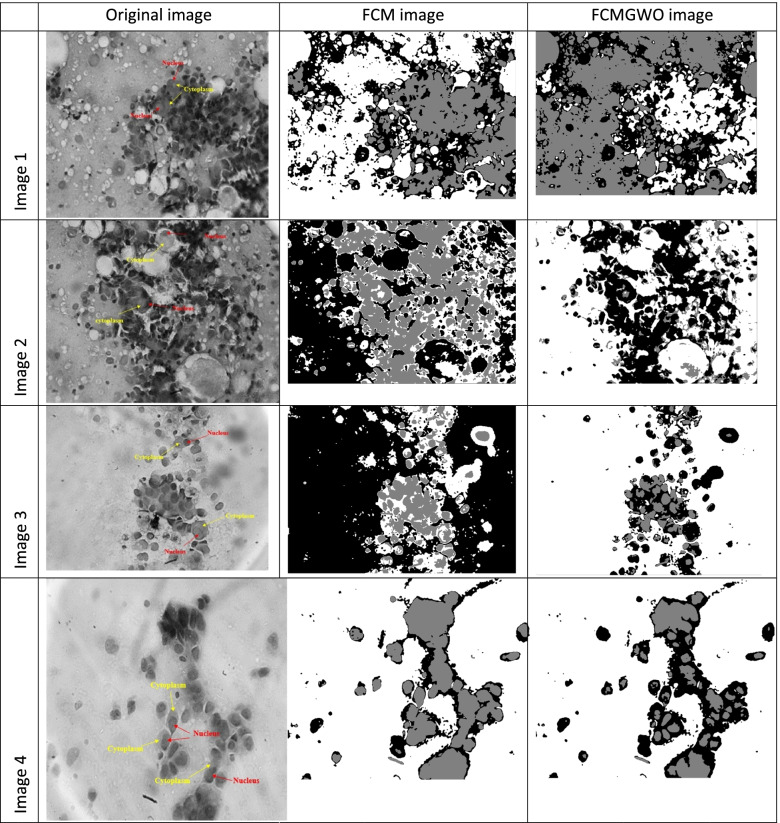
Cytology images based on original and segmented using FCM and FCMGWO methods. The original images are samples of the frozen section of Breast cancer from a 50-year-old woman. The diagnostic result is invasive ductal carcinoma with a score of 7.9 and grade II/III. The tumor size is 5.5 × 5 × 3. The original images were first resized to 800 × 600 pixels. Nucleus with red color and cytoplasm with yellow color were labelled

**Fig. 2:**
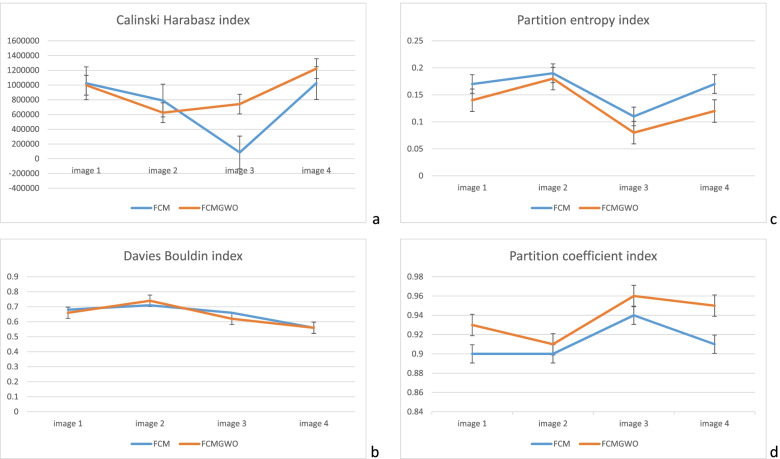
Validation indexes for comparing FCM and FCMGWO. **a** Calinski Harabasz index (CH) **b** Davies Bouldin index (DB) **c** Partition entropy index (Vpe) **d** Partition coefficient index (Vpc). The FCM and FCMGWO methods have a significant difference with respect to the Vpc and Vpe indices. However, there is no significant difference between the performances of the two clustering methods with respect to the Calinski-Harabasz and Davies-Bouldin indices

The image segmentation results can be seen in Fig. 1. In all the analyzed images, FCMGWO performed better than FCM in separating the nucleus from the other dark objects in the cell. The points corresponding to the nucleus and other dark objects, such as cytoplasm and red blood cells, have been considered as one cluster by FCM. In FCMGWO, however, these points have been designated as the nucleus, and the other objects have been distinguished as two clusters. The performances of the FCMGWO and FCM in segmenting cytology images were compared. The performance of the algorithms was evaluated using V_pc_, V_pe_, DB, and CH validation indices. The clustering result is acceptable when V_pc_ and CH are maximum and V_pe_ and DB are minimum (Fig. 2). A study of the indices presented in Fig. 2 reveals the superiority of FCMGWO over FCM with V_pe_ and V_pc_ criteria for all images. According to the CH index, FCMGWO is better than FCM for images 3 and 4, while FCM is better than FCMGWO for images 1 and 2.

The paired t-test was used to compare the significance between the indices. The normality of the V_pc_, V_pe_, DB, and CH indices was examined using the Shapiro–Wilk test (*p* -value > 0.05). Then, the paired t-test was used to investigate the significance of the differences in the indices (Table 1). The FCM and FCMGWO methods have a significant difference with respect to the V_pc_ and V_pe_ indices. However, there is no significant difference between the performances of the two clustering methods with respect to the DB and CH indices.

**Table 1:** Paired t-test results indicating the difference between the performance of the indices

*P*-value	T	Methods	Index
0.030	-3.873	FCM:FCMGWO	V_pc_
0.035	3.674	FCM:FCMGWO	V_pe_
0.650	0.502	FCM:FCMGWO	DB
0.426	-0.920	FCM:FCMGWO	CH

## Discussion

Changes in the structure of the nucleus are the morphological hallmark of cancer diagnosis and most of the criteria of malignancy are seen in the nuclei of the cell. Therefore, it is necessary to separate the nuclei from other parts of the image. The segmentation of images containing objects with similar color intensity is a challenge in image processing. It is difficult to distinguish the cell nucleus from other cell components, such as red blood cells and plasma, in cytology images due to color similarities. The current study proposed the FCMGWO method for this purpose. This technique was compared to the FCM and validated for breast cytology images.

The results indicate that FCM is incapable of identifying the cell nucleus. FCM considers the nucleus and other dark objects in the cell as one cluster and cannot distinguish between them. However, the combined FCMGWO method performs better than FCM in distinguishing the cell nuclei. This better discernment can be due to the search process of the GWO, which optimizes the cluster centers obtained from FCM clustering. This optimization can improve the performance of FCM and overcome some of its shortcomings owing to its high exploration capability and the good agreement between the exploration and exploitation in GWO.

The improvement in V_pc_ and V_pe_ using FCMGWO is more statistically significant compared to FCM. However, no significant difference was observed between the DB and CH indexes using the two clustering methods. Based on the CH index, FCMGWO performs better than FCM for images 3 and 4, but FCM is better than FCMGWO for images 1 and 2. The CH index also shows that FCMGWO is better than FCM in images with less detail. However, the DB indices of the two methods are almost identical without differences in image type. Lack of ground truth was our main limitation in this study. Therefore, it is not possible to compare clustering results with indices such as sensitivity and specificity.

In future studies, the algorithm can be tested on other images with similar color intensity. Furthermore, the overall performance of the proposed method in images with more detail can be improved by a fuzzy algorithm modified via adding a more powerful objective function.

## Conclusion

The results show that the FCMGWO method performs better on images with less detail than those with more detail. The hybrid algorithm distinguishes the cells better in images with less detail than in images with high detail. However, FCM exhibits unacceptable performance in both low- and high-detail images.

## Methods

The image analysis consists of preprocessing and segmentation steps, which will be discussed in detail in subsequent sections.
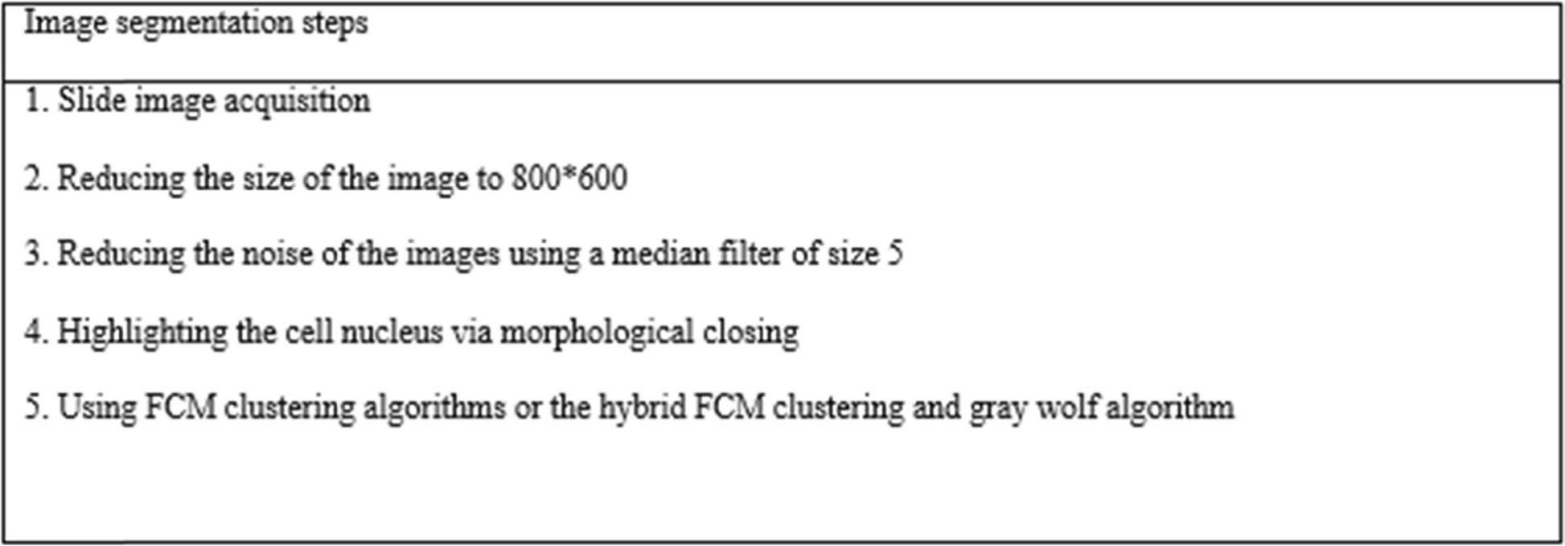


### Actual images

Imprint touch breast cytology images were utilized to examine the segmentation methods. All the images were confirmed by a pathologist. The images were produced with a magnification of 400x.

**Table:** 

Histological preparation and staining
Preparing a smear
Alcohol 96% (time: 1 min or 30 s)
Preferably 80% alcohol
Rinse with a gentle stream of water
Hematoxylin for a few moments
Wash
Lithium carbonate
Rinse so that the lithium carbonate remains on the slide surface
Eosin just a few moments
Wash
Alcohol 70%, 96% and 100% (stops more in 100% alcohol)
Xylene
Montage

The image for digital analysis was generated by a echoLAB camera mounted atop an echoLAB microscope. They were first resized to 800*600 to reduce the processing time. All the analyses were performed using Python 3.8 and SPSS 26.

### Image preprocessing

Image noise is the random change in the brightness or color data of an image [7] and can severely deteriorate image quality [8]. In addition to denoising, preserving the edges and details of an image plays a key role in image processing [8]. In this study, a median filter of size 5 is used to reduce the noise from the camera. After using the median filter, morphological closing is employed to highlight the nucleus of the cell in the images.

### Image segmentation

After preprocessing, image segmentation was performed via clustering techniques. Classification of tissue as malignant or benign requires detecting the nucleus in the cytology images. This is a challenging task since the images usually contain overlapping and clustered objects. In this study, FCM and FCMGWO clustering were used for image segmentation.

### FCM clustering

FCM is a powerful unsupervised method for data analysis. This technique is most widely used in image segmentation [9]. FCM aims to divide the data inside the subspaces according to the distance criterion [5]. The objects at the boundaries between different classes do not have to belong fully to one class but are rather assigned membership degrees between 0 and 1 [9]. FCM clustering was introduced by Bezdek in 1973. The objective function of FCM is defined as follows [10].

$${J}_{m}=\sum_{i=1}^{n}\sum_{j=1}^{c}{u}_{ij}^{m}{\Vert {x}_{i}-{c}_{j}\Vert }^{2}$$

where m represents the degree of fuzziness and is a real number greater than 1, u_ij_ is the membership degree of the i^th^ datum in the j^th^ cluster, x_i_ denotes the data points, and c_j_ is the cluster center. Also, $\Vert \bullet \Vert$ represents the Euclidean distance, n is the number of data points, and c denotes the number of clusters [11].

The initial parameters were initialized as follows: Number of clusters = 3; Fuzziness factor = 1.5; Number of iterations = 5.
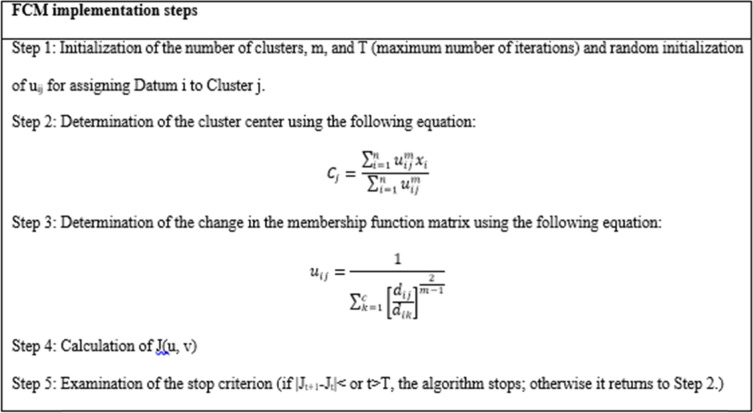


### Gray wolf optimization

Optimization is a common method in machine learning for searching for the best solution or a sufficiently good solution. The GWO is a heuristic swarm intelligence optimization algorithm introduced by Mirjalili et al. in 2014 [6]. The best, second-best, and third-best responses are recorded as alpha, beta, and delta, respectively, and the rest of the wolves are considered as omega [12]. Optimization algorithms require exploration and exploitation in a search space. In GWO, exploration refers to when the wolf leaves the initial search path in a specific context and turns to a new direction [12]. Exploitation refers to when the wolf searches more accurately in the initial search path in a specific context [12]. An optimization algorithm requires a good agreement between the exploration and exploitation steps for successful implementation [13]. GWO has a high exploration capability in finding the best solution for the problem. This capability prevents the entrapment of the algorithm in local optima [6].

### Mathematical modeling of GWO

#### Encircling the prey

Gray wolves encircle the prey during hunting. The following equations are proposed to model the encirclement behavior [6]:

$$\overrightarrow{D}=\left|\overrightarrow{C}*{\overrightarrow{X}}_{p}(t)-\overrightarrow{X}(t)\right|$$

$$\overrightarrow{X}\left(t+1\right)={\overrightarrow{X}}_{p}\left(t\right)-\overrightarrow{A}*\overrightarrow{D}$$

where

$$\overrightarrow{A}=2\overrightarrow{a}*{\overrightarrow{r}}_{1}-\overrightarrow{a}$$

$$\overrightarrow{C}=2*{\overrightarrow{r}}_{2}$$

where t is the current number of iterations, $\overrightarrow{A}$ and $\overrightarrow{C}$ are the coefficient vectors, ${\overrightarrow{X}}_{p}$ is the position vector of the prey, and $\overrightarrow{X}$ is the position vector of the gray wolf [6]. D is the distance between the positions of the prey and the wolf at time t. The components $\overrightarrow{a}$ decrease linearly from 2 to 0 during the iteration, and r_1_ and r_2_ are random vectors in the range [0,1] [6].

#### Hunting

Gray wolves are able to identify the hunting location and encircle them. However, there is no idea of the optimal position of the prey in a search space [6]. It is assumed that the alpha, beta, and delta have more knowledge about the potential position of the prey [6]. Therefore, the first three obtained solutions are stored, and the other agents are responsible for updating their positions according to that of the best search agent [6]. The following formulae are presented in this regard [6]:

$${\overrightarrow{D}}_{\alpha }=\left|{\overrightarrow{C}}_{1}\bullet {\overrightarrow{X}}_{\alpha }-\overrightarrow{X}\right|, {\overrightarrow{D}}_{\beta }=\left|{\overrightarrow{C}}_{2}\bullet {\overrightarrow{X}}_{\beta }-\overrightarrow{X}\right|, {\overrightarrow{D}}_{\delta }=\left|{\overrightarrow{C}}_{3}\bullet {\overrightarrow{X}}_{\delta }-\overrightarrow{X}\right|$$

In updating, a hypothetical position must be considered for the prey since the position of the prey is unknown. The best option for this hypothetical position is the best position the wolves have been at so far. The position ${\overrightarrow{X}}_{p}(t)$ must be replaced by those of the alpha, beta, and delta wolves, and D_α_, D_β_, and D_δ_ must be calculated as the distances between these wolves and the prey, respectively.

$${\overrightarrow{X}}_{1}={\overrightarrow{X}}_{\alpha }-{\overrightarrow{A}}_{1}\bullet \left({\overrightarrow{D}}_{\alpha }\right), {\overrightarrow{X}}_{2}={\overrightarrow{X}}_{\beta }-{\overrightarrow{A}}_{2}\bullet \left({\overrightarrow{D}}_{\beta }\right) , {\overrightarrow{X}}_{3}={\overrightarrow{X}}_{\delta }-{\overrightarrow{A}}_{3}\bullet ({\overrightarrow{D}}_{\delta })$$

$$\overrightarrow{X}\left(t+1\right)=\frac{{\overrightarrow{X}}_{1}+{\overrightarrow{X}}_{2}+{\overrightarrow{X}}_{3}}{3}$$

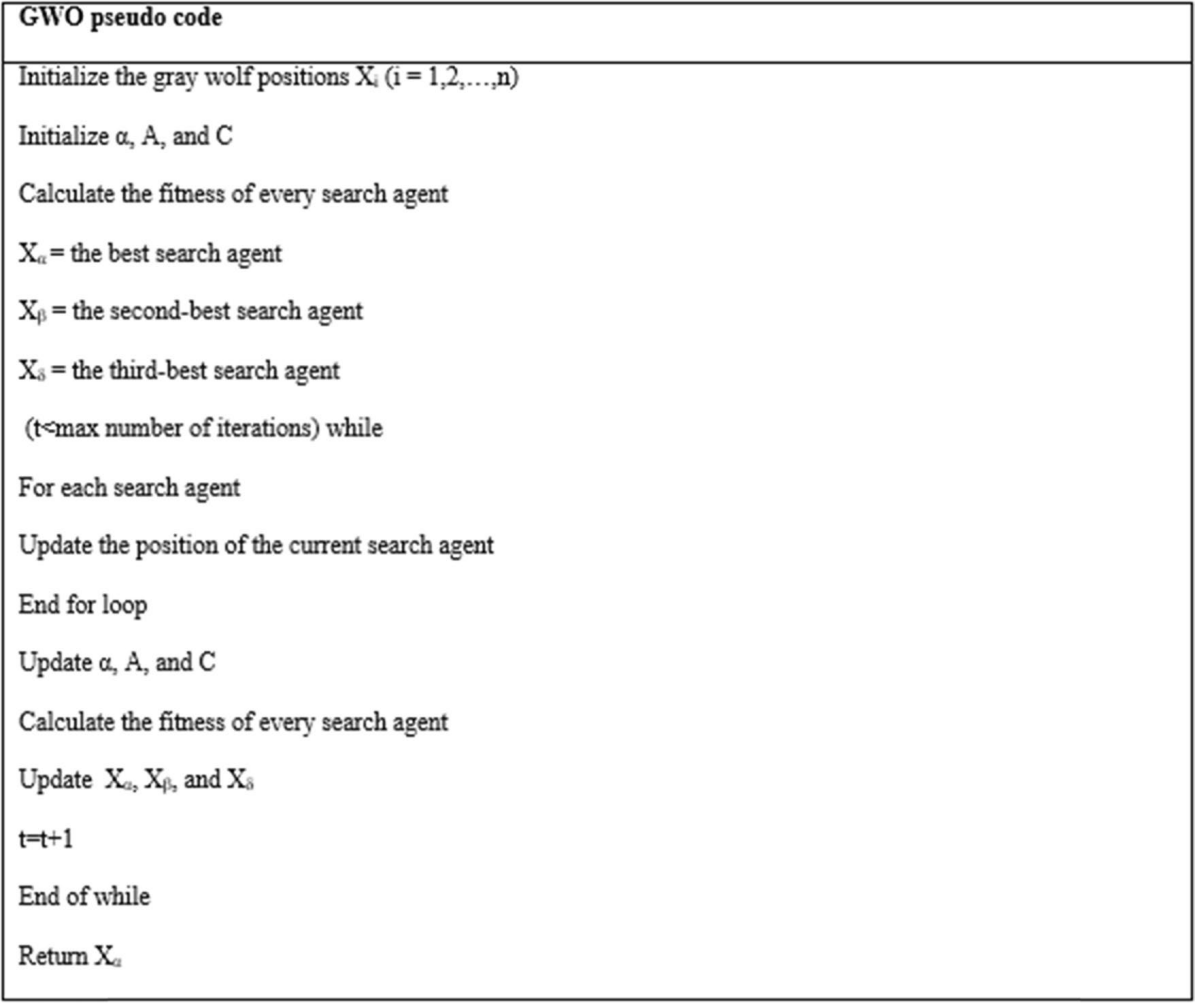


#### Proposed method

The proposed segmentation method is a technique that combines the benefits of FCM and GWO. FCM is a recursive algorithm that aims to find the cluster centers such that the dissimilarity function is minimized. The FCM-GWO method was employed for the segmentation of images in order to counterbalance the drawbacks of FCM clustering. The cluster centers obtained from FCM are input to the GWO algorithm as initial positions to improve the FCM results and better distinguish the cell nucleus. The details of this hybrid technique are expressed in the following algorithm.

The parameters were initialized as follows: Number of clusters = 3; Number of search agents = 5; Fuzziness factor = 1.5; Number of iterations = 5; Lower bound of the search space = 0; Upper bound of the search space = 225.
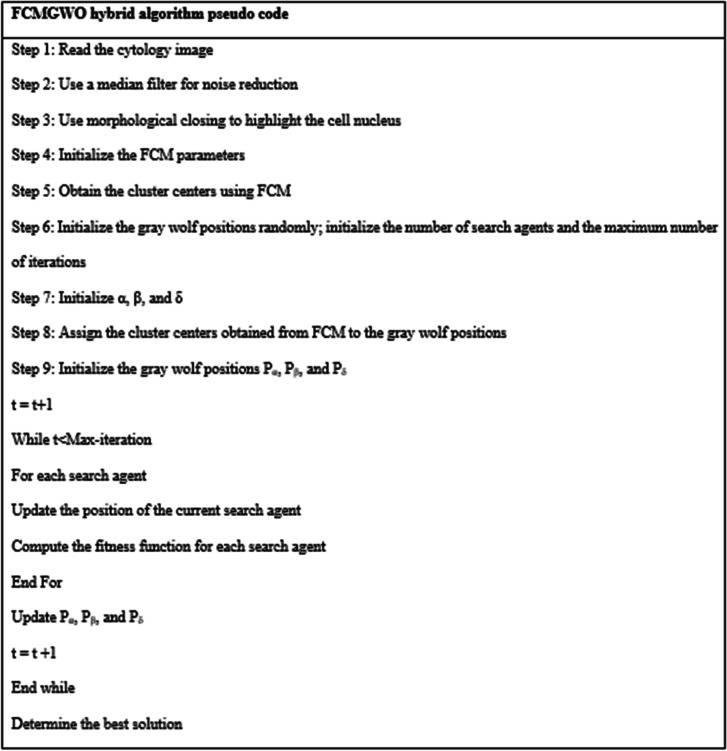


#### Validation of the clustering methods

Two groups of indexes were introduced to evaluate the clustering algorithms. The first group uses only the membership values (u_ij_), while the second group uses the membership matrix and the data [14]. The partition coefficient (PC) and the partition entropy (PE) coefficient variance indices were selected from the first group, and the Calinski-Harabasz (CH) and Davies-Bouldin (DB) indices were chosen from the second group [14].

#### Validity indices

V_PC_ and V_pe_ are in the range [0,1], and optimal clustering has been obtained when V_PC_ is maximum or V_pe_ is minimum. DB index is in the range (0, ∞). A lower value represents better clustering. A higher CH value represents better clustering.

## Data Availability

The datasets used and/or analyzed during the current study available from the corresponding author on reasonable request.

## Abbreviations

FCM: Fuzzy c-means
GWO: Gray wolf optimization
CH: Calinski-Harabasz
DB: Davies-Bouldin
V_pe_: The partition entropy coefficient variance
V_pc_: Partition coefficient
